# Prevention of white spot lesions with fluoride varnish during orthodontic treatment with fixed appliances: a systematic review

**DOI:** 10.1093/ejo/cjad013

**Published:** 2023-04-10

**Authors:** Mikael Sonesson, Svante Twetman

**Affiliations:** Department of Orthodontics, Faculty of Odontology, Malmö University, Malmö, Sweden; Department of Odontology, Faculty of Health and Medical Sciences, University of Copenhagen, Copenhagen, Denmark

## Abstract

**Background:**

Fluoride varnish (FV) is an established technology for primary and secondary caries prevention.

**Objective:**

The aim of this review was to evaluate the preventive effect of FV on development of white spot lesions (WSL) when regularly applied during orthodontic treatment with fixed appliances.

**Search methods:**

We searched PubMed, Scopus and Google Scholar up to October 2022 using predetermined keywords.

**Selection criteria:**

We included randomized controlled trials of a duration of minimum 12 months and at least quarterly FV applications.

**Data collection and analysis:**

Based on abstracts, we retrieved full-text papers, extracted key outcome data, and assessed risk of bias. Primary outcome was prevalence of WSLs on subject level after debonding. We conducted a narrative synthesis and pooled comparable outcome data in a random effects model.

**Results:**

We included seven studies covering 666 patients and assessed four publications with low or moderate risk of bias and three with high. The prevalence of WSLs at debonding varied between 12 and 55%. All studies presented results in favour for the FV intervention, one reached statistical significance on subject level. Five studies provided data for a meta-analysis. The pooled risk ratio was 0.64 [95% CI: 0.42, 0.98], indicating a statistically significant preventive effect. Certainty of evidence was graded as very low after reducing for risk of bias, inconsistency and imprecision.

**Limitations:**

We pooled data on subject level and did not consider lesion severity on tooth level.

**Conclusions and implications:**

Even if the certainty of evidence was very low, it was shown that FV can prevent development of WSL when regularly applied during orthodontic treatment. Larger investigations reporting a core outcome set are required to increase the certainty of evidence.

**Registration:**

PROSPERO database (CRD42022370062).

## Introduction

Treatment with fixed multi-bracket orthodontic appliances is associated with impaired oral hygiene and an ecological perturbation of the biofilm adjacent to the bracket base (1, 2). This may result in dysbiotic conditions and enamel mineral loss, clinically characterized as opaque, and rough white areas of enamel appearing along the bracket base. Although the prevalence of such white spot lesions (WSL) is high at the time point of bracket removal (20–50%), many of the minor lesions will reverse within the first year after debonding (3). The more advanced WSLs are however clearly visible after several years and may thereby jeopardize the aesthetic outcome of the orthodontic treatment. Therefore, the best clinical practice would be to secure topical preventive measures during the course of the multi-bracket orthodontic treatment. Several systematic reviews have addressed this topic (4–7) and the best available evidence for reducing the risk of enamel demineralization comes from professional and self-applied fluorides (4). In pediatric dentistry practice, fluoride varnish (FV) is a well-proven technology for primary and secondary caries prevention in the young permanent dentition when applied quarterly or bi-annually (8). However, the benefits in adjunct to daily tooth cleaning with fluoride toothpaste brushing has recently been questioned (9).

Since the most recent Cochrane review on the prevention of enamel demineralization during fixed brace treatment was published (4), a number of new clinical trials have been published, indicating a high activity in this field of research. Systematic reviews must be periodically updated in order to guide clinicians on the best available scientific evidence for a given intervention. In this paper, the focused research question was ‘*How effective are fluoride varnish in preventing white spot lesions when regularly applied during orthodontic treatment with fixed appliances*?’ The PICO was: **Population**—healthy adolescents and adults undergoing orthodontic multi-bracket treatment; **Intervention**—periodic applications of FV around the bracket bases; **Control**—placebo varnish applications, other comparisons or standard care; **Outcome**—prevalence or incidence of WSL at debonding, assessed with any clinically validated index.

## Evidence acquisition

### Search methods for identification of studies

This review followed the PRISMA statement (10) and the protocol was registered in the PROSPERO database (CRD42022370062). We searched the electronic databases PubMed and Scopus up to October 7 2022 for relevant literature and the search strategies are provided in Table 1. In addition, we applied the search terms in Google Scholar as a complement. The eligibility criteria were peer-reviewed publications in English, reporting a randomized controlled trial with parallel groups and a duration of a minimum of 12 months. We required at least quarterly topical applications of the varnish around the bracket bases and an outcome reported by a clinical index with predefined scores (categorized data). Reports based on continuous data such as laser fluorescence and quantitative light-induced fluorescence were not accepted. We also excluded studies based on a split-mouth design, and single- or bi-annual varnish applications. In addition, we disregarded studies based on convenience samples, case series, case descriptions, narrative reviews, and grey literature, such as textbooks, conference papers, monographs, and thesis. The reference lists of all selected papers, including available systematic reviews, were hand-searched for possible additional references. We searched www.clinicaltrials.gov to identify registered ongoing studies by combining the phrase ‘FV’ with ‘orthodontics’ and/or ‘fixed appliances’.

**Table 1.** Search strings in (A) PubMed via NLM, and (B) Scopus via Elsevier, October 2022.

		Result
(A) Topical fluorides		
1	‘Fluorides, Topical’[Mesh] OR topical fluorides OR fluoride varnish OR fluoride lacquer	6152
Early caries		
2	‘Dental Caries’[Mesh] OR dental caries OR white spot OR early caries OR initial caries OR enamel caries OR carious lesions OR carious lesion	69 920
Orthodontics		
3	‘Orthodontics’[Mesh] OR orthodontic appliances OR orthodontic treatments OR fixed appliances OR multi-bracket treatment OR multi-bracket appliances OR orthodontic bracket OR orthodontic brackets OR orthodontic braces OR permanent appliances OR fixed retainers OR fixed retainer OR permanent retainer OR permanent retainers OR dental braces OR fixed braces OR permanent braces	83 728
4	1 AND 2 AND 3	297
(B) Topical fluorides		
1	ALL(‘topical fluorides’ OR ‘fluoride varnish’ OR ‘fluoride lacquer’)	11 626
Early caries		
2	ALL(‘dental caries’ OR ‘white spot’ OR ‘early caries’ OR ‘initial caries’ OR ‘enamel caries’ OR ‘carious lesions’ OR ‘carious lesion’)	126 549
Orthodontics		
3	ALL(‘orthodontic appliances’ OR ‘orthodontic treatment’ OR ‘fixed appliances’ OR ‘multi bracket treatment’ OR ‘multi bracket appliances’ OR ‘orthodontic bracket’ OR ‘orthodontic brackets’ OR ‘orthodontic braces’ OR ‘permanent appliances’ OR ‘fixed retainers’ OR ‘fixed retainer’ OR ‘permanent retainer’ OR ‘permanent retainers’ OR ‘dental braces’ OR ‘fixed braces’ OR ‘permanent braces’)	61 566
4	1 AND 2 AND 3	1370

### Selection of studies

**Figure 1:**
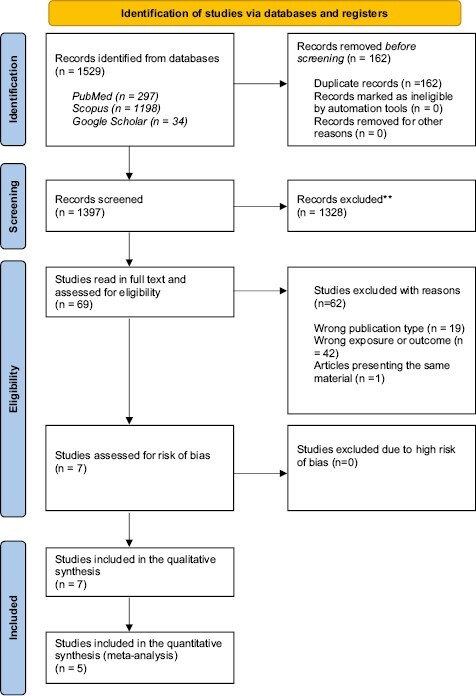
PRISMA 2020 flow chart showing the selection of studies included in this review.

The search process gave 1529 unique hits. After removal of duplicates and obviously irrelevant titles, two authors (ST, MS) assessed the abstracts of potentially eligible studies independently and ordered full-text papers for further evaluation of relevance. We show a flowchart of the study selection in Figure 1. The excluded publications are listed in Supplementary Table S1 together with the main reason for the exclusion.

### Data extraction and management

The two authors extracted data from the included studies independently from each other. We tabulated the following items: first author, year of publication, country of origin, age of study group, number of subjects in test and control groups, duration of the study and index used for WSL scoring. In addition, we identified the type of FV, the frequency of the applications, as well as the control group interventions. The primary outcome measure was the prevalence of WSL on subject level immediately after debonding of brackets, expressed as percent. If data were unclear or missing, we contacted the corresponding author by e-mail for clarifications.

### Assessment of risk of bias

The authors assessed the risk of bias (low, moderate, high) independently for each study according to the handbook of Swedish Agency for Health Technology Assessment and Assessment of Social Services (11). We solved disagreements with a consensus discussion with a third independent reviewer.

### Data synthesis

The authors conducted a narrative synthesis of the included studies. For studies with comparable outcome measures, we pooled data in a random effects model using the Review Manager 5.3 tool (The Nordic Cochrane Center, Copenhagen, Denmark). The certainty of evidence was expressed according to the GRADE working group (12).

### Ethics

Ethical approval was not applicable for present study.

## Results

### Study characteristics

We included seven randomized controlled trials conducted in Sweden, Poland, Iran, Hong Kong and the United States covering a total of 666 patients (Tables 2 and 3). Most of them were adolescents and young adults, although the age range varied from 12 to 50 years. Three studies evaluated a 5% sodium FV (13–15), two a 5% sodium FV with CPP-ACP (16, 17), while a 0.1 difluorosilane varnish (18) and a 1.5% ammonium FV (19) was tested in one study each. Three studies were placebo controlled (13, 18, 19), one applied resin sealants on control teeth (17) and three studies had ‘standard care’ with oral hygiene instructions in the control group (14–16). The frequency of applications ranged from every 4th week to once every 3rd month, and the duration of the projects varied between 12 and 26 months. All seven studies reported the incidence or prevalence of WSLs at debonding on subject or tooth level aid of categorized scores. In four trials, the scoring was conducted from clinical photographs (14, 17–19). Four papers described an intra- or inter-examiner validation process associated with the outcome measure (16–19). The presence or absence of side effects/adverse events was addressed in three of the publications (13, 18, 19). In total, one patient reported nausea and aborted the intervention while five patients complained over taste and discoloration.

**Table 2.** Main characteristics of the included studies.

First author, year/country	Age	Test/control	Duration	Dropout (%)	WSL score
Stecksen-Blicks, 2007/Sweden	12–15 years	137/136	Mean 1.7 years	5.00	Gorelick/photo
Rechmann, 2018/USA	13–26 years	20/20	12 months	7.50	EDI/clinical
Sonesson, 2020/Sweden	12–18 years	85/81	Mean 1.7 years	10.80	Gorelick/photo
Zarif Najafi, 2021/Iran	12–18 years	30/30	12 months	3	Gorelick/clinical
Flynn, 2022/USA	12–17 years	20/20	12 months	0	EDI/photo
Grocholewicz, 2022/Poland	16–50 years	30/30	12 months	0	Gorelick/clinical
Sardana, 2022/Hong Kong	13–25 years	33/33	18 months	10.60	Gorelick/photo

**Table 3.** Intervention and main results on subject level of the included studies.

First author, year	Test^#^	Control	Frequency/applic. (*n*)	WSL prevalence T%/C%	WSL incidence T%/C%	*P*
Stecksen-Blicks, 2007	0.1% DFS	Placebo	6 weeks (*n* = 10)	12/30	7/26	<0.05
Rechmann, 2018	5% NaF+MI^A^	Standard care^B^	3 months (*n* = 4)	54/55^C^	32/41^C^	NS
Sonesson, 2020	1.5% AmF	Placebo	6 weeks (*n* = 13)	42/44	12/26^D^	NS/^D^<0.05
Zarif Najafi, 2021	5% NaF	Placebo	3 months (*n* = 4)	23/32^C^	NR	<0.05
Flynn, 2022	5% NaF+MI	Sealants	4–6 weeks (*n* = 8–12)	35/50	35/50	NS
Grocholewicz, 2022	5% NaF	Standard care	4 weeks (*n* = 12)	17/27	17/27	NS
Sardana, 2022	5% NaF	Standard care	4 weeks (*n* = 18)	10/18	NR	NS

NR, not reported; NS, not statistically significant.

^A^Subjects were recommended to use a 0.05% sodium fluoride mouth rinse daily.

^B^Topical application of a CPP-ACP paste (900 ppm F) once daily after brushing.

^C^Data reported as percentage of teeth.

^D^Incidence of advanced WSLs only (score 3 + 4).

^#^DFS, difluorosilane varnish; NaF, sodium fluoride varnish; NaF+MI, sodium fluoride varnish + 10% casein phosphopeptide-amorphous calcium phosphate; AmF, ammonium fluoride.

### Risk of bias within studies

**Figure 2:**
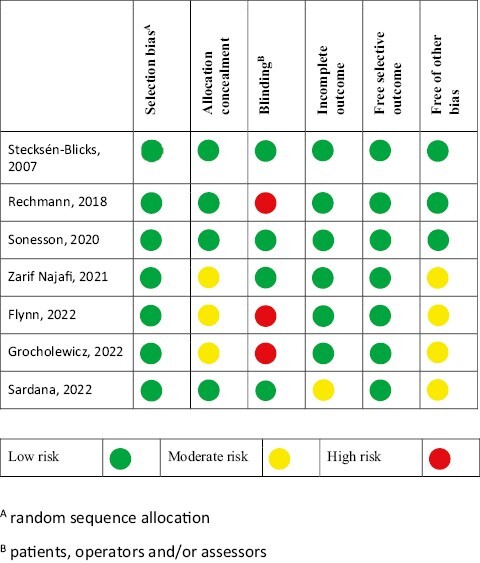
Risk of bias item for each included study.

Figure 2 displays the risk of bias of the included studies. We assessed four studies at low or moderate risk of bias (13, 14, 18, 19), and three with high risk of bias due to lack of blinding (15–17).

### Results of individual studies

Results on subject level was available from five studies while two reported data on tooth level (13, 16). All studies presented results (WSL prevalence at debonding, or incidence of WSLs during orthodontic treatment) more or less in favour for the FV intervention but only one reached statistical significance on patient level (18). Two trials presented statistically significant differences on tooth level (13, 18).

### Synthesis of results

**Figure 3:**
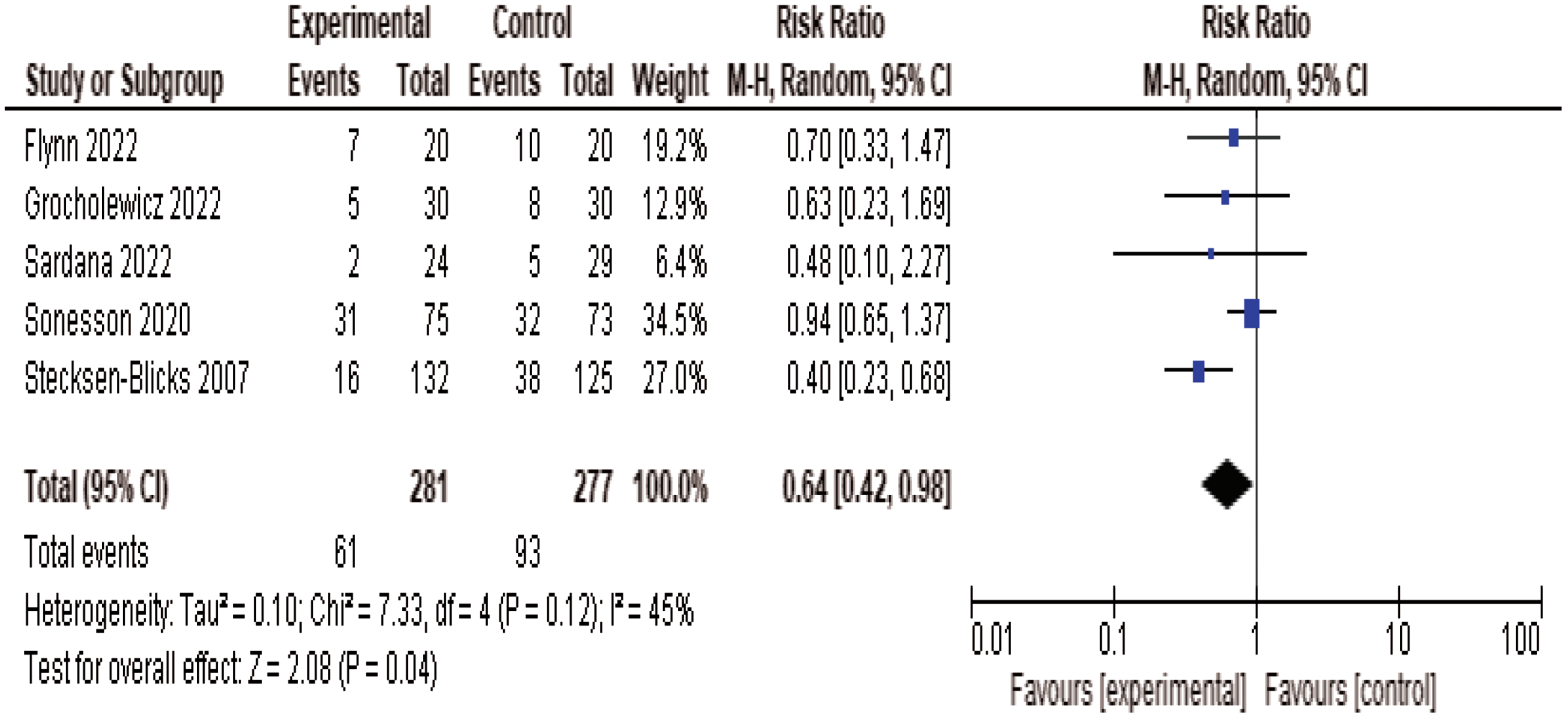
Forest plot showing pooled effect of regular fluoride varnish applications during treatment with fixed orthodontic appliances on the prevalence of white spot lesions at debonding. Note that the outcome in the study of Sardana et al. (14) was measured 18 months after the application of brackets.

We pooled the prevalence of WSLs immediately after debonding of brackets on subject level from five studies in a meta-analysis and the forest plot is presented in Figure 3. Using a random effects model, the risk ratio was 0.64 (95% CI: 0.42, 0.98) demonstrating a favourable effect of the FV intervention (*P* < 0.05). The *I*^2^ value of 45% indicated that the studies represented a moderate heterogeneity. Two studies reported the clinical scores as mean values (18, 19). The standard mean difference between the test and control groups based on 258 patients was −0.44 (−0.64, −0.24; *P* < 0.05).

### Certainty of evidence

The certainty of evidence was graded as very low after reducing for risk of bias (−1), inconsistency (−1) and imprecision (−1).

## Discussion

The main finding of this systematic review was that regular professional applications of a fluoride during treatment with fixed multi-bracket orthodontic appliances is associated with a reduced prevalence of WSL at the time of debonding. We analyzed the primary outcome on subject level in order to secure independent units, which enabled us to merge five trials. The estimated relative risk was of the same magnitude as previously reported for young permanent teeth in pediatric dentistry care (8) and among orthodontic patients (20). An interesting observation was that three of the most recent trials reported that the protective effect of the varnish was most explicit for the advanced lesions (13, 14, 19). Prevention of the advanced and cavitated WSLs is a priority since they normally require further treatment in terms of resin infiltration, micro-abrasion, bleaching, or fillings in order not to compromise the final esthetic result (21). It is however important to bear in mind that neither the placebo or standard care control groups in this review were deprived from fluoride exposure. Oral hygiene instructions were given to all participants, and they were strongly urged to brush their teeth twice daily with an adult (1450 ppm) fluoride toothpaste. In one study, the subjects of the control group were even encouraged to use a 0.05% sodium fluoride mouth rinse on daily basis (16).

None of the studies provided health-economic aspects of the intervention. Two studies reported the number needed to treat, ranging from 5.5 to 7.1. This implies that the FV intervention may be cost saving, in particular when performed by dental auxiliaries in connection to the regular appliance adjustments. Ideally, FV treatment should also be personalized, based on the individual caries risk assessment before onset of the appliances and the ability to maintain a high level of oral hygiene during the multi-bracket treatment.

In this review, we excluded reports with continuous endpoints based on digital technologies, such as laser fluorescence and Digital Imaging Fiber-Optic TransIllumination (DiFOTI). Such measurements might indeed be very useful in clinical trials investigating the management of post-orthodontic WSLs in particular, but the usefulness during ongoing multi-bracket treatments lack validation. We included studies that scored the endpoint with either the Gorelick index or the enamel decalcification index, both based on four comparable categories. In four studies, the WSL scoring was made from clinical photographs which is considered as a reproducible and reliable method for assessment of such lesions (22). Nevertheless, in harmony with Wang and co-workers (23) we found a substantial heterogeneity in reporting the outcome among the included studies. The incidence of WSLs during treatment was alternatively reported on subject level or tooth level with mean values, frequency figures or absolute numbers. In other papers, only the prevalence of WSLs at the time of debonding was presented, assuming that teeth were free from enamel demineralization at the onset of appliances. This variation hampered the pooling of the results, and we contacted the authors for additional data, unfortunately without success. Moreover, none of our selected studies reflected the patient’s perspectives on the intervention and its adverse effects. Therefore, the development of a core outcome set for trials on the prevention and treatment of orthodontically induced enamel WSL (24) will certainly facilitate and enhance future studies in this area. It is however not enough to harmonize just what, when and how to measure; also the patients perspective should also be taken into account.

We assessed three of the seven studies with high risk of bias. The most common item of concern was the lack of blinding of clinicians and patients followed by an unclear allocation concealment. Some of the studies were small and thereby possibly underpowered, in particular concerning the less-frequent advanced lesions. One reason for this shortage was that three trials employed an ambitious three- (14) or four-arm parallel design (13, 15) investigating multiple interventions. We extracted data from the intervention groups that were most relevant for the present research question, but notable, six different brands of FV were employed in the seven selected studies. A possible confounder for the in-between study comparisons was that a flavoured FV with bio-available calcium and phosphate was applied in two studies. Although indicated for hypersensitive and hypomineralized teeth, the 5% NaF component justified us to merge the caries-protective effects with the standard varnishes without calcium and phosphate. For future RCTs, we suggest that important key issues are addressed; 1. Sufficient number of patients with independent allocation concealment; 2. Blinded assessments on patient and tooth level, 3. Use of a validated core outcome set; and 4. Patient perceptions, during and after the intervention. In addition, the implementation of WSL-preventive technologies in the everyday orthodontic care needs further attention.

## Conclusions

Regular professional applications of a FV during treatment with fixed orthodontic appliances can prevent the development of WSL adjacent to brackets but the certainty of evidence is very low. Thus, further research is warranted to elucidate the clinical efficiency, health-economic aspects and patients perceived value of FV applications in orthodontic practice.

## Supplementary Material

cjad013_suppl_Supplementary_File_S1

## Data Availability

The data that support the findings of this systematic review are available from the corresponding author, upon reasonable request.
