# Supplementary material for: Prevention of white spot lesions with fluoride varnish during orthodontic treatment with fixed appliances: a systematic review
Source: Eur J Orthod. 2023 Apr 10;45(5):485–90. doi: 10.1093/ejo/cjad013 (PMC10505687; doi:10.1093/ejo/cjad013)
Supplement: cjad013_suppl_Supplementary_File_S1 [file cjad013_suppl_supplementary_file_s1.docx]

**Supplementary file S1: Excluded studies**

______________________________________________________________________________

First author, year Main reason for exclusion

______________________________________________________________________________

Abufarwa 2022 in vitro study

Alavi, 2018 small sample size, adult patients

Baygin, 2013 single fluoride varnish application

Demito, 2011 split mouth design, few patients

Farhadian, 2008 inadequate endpoint

Giray, 2018 not orthodontic patients

Hamdan, 2018 in vitro study

Kirschneck, 2018 single fluoride varnish application

Knösel, 2012 in vitro study

Mehta, 2015 inadequate endpoint

Øgaard, 2001 lack relevant control group, fluoride varnish in all test groups

Perrini, 2016 split-mouth design, few patients

Poornima, 2021 inadequate endpoint

Restrepo, 2016 secondary prevention, two applications on active lesions

Shah, 2018 split-mouth design, single fluoride varnish application

Shinaishin, 2011 inadequate endpoint

Silva, 2021 inadequate endpoint

Vivaldi-Rodrigues, 2006 split-mouth design, few participants

______________________________________________________________________________

**References – excluded studies**

Abufarwa, M., Noureldin. A., Dziak, R. and Covell, D. (2022) Efficacy of CPP-ACP fluoride varnish applied with and without acid etching in preventing enamel demineralization compared to light-curable fluoride varnish. *Angle Orthodontist*, 1, 213-219. doi: 10.2319/050121-345.1.

Alavi, S., and Yaraghi, N. (2018) The effect of fluoride varnish and chlorhexidine gel on white spots and gingival and plaque indices in fixed orthodontic patients: A placebo-controlled study. *Dental Research Journal (Isfahan)*, 15, 276-282.

Baygin, O., Tuzuner, T., Ozel, M.B. and Bostanoglu, O. (2013) Comparison of combined application treatment with one-visit varnish treatments in an orthodontic population. *Medicina Oral, Patologia Oral, Cirugia Bucal*, 1, e362-370.

Demito, C.F., Rodrigues, G.V., Ramos, A.L. and Bowman, S.J. (2011) Efficacy of a fluoride varnish in preventing white-spot lesions as measured with laser fluorescence. *Journal of Clinical Orthodontics*, 45, 25-29.

Farhadian, N., Miresmaeili, A., Eslami, B. and Mehrabi, S. (2008) Effect of fluoride varnish on enamel demineralization around brackets: an in-vivo study. *American Journal of Orthodontics and Dentofacial Orthopaedics*, 133, 4 Suppl., S95-98.

Giray, F.E., Durhan, M.A., Haznedaroglu, E., Durmus, B., Kalyoncu, I.O. and Tanboga, I. (2018) Resin infiltration technique and fluoride varnish on white spot lesions in children: Preliminary findings of a randomized clinical trial. *Nigerian Journal of Clinical Practice*, 21, 1564-1569.

Hamdan, W.A., Badri, S, El Sayed, A. (2018) The effect of fluoride varnish in preventing enamel demineralization around and under orthodontic bracket. *International Orthodontics*, 16, 1-11.

Kirschneck, C., Christl, J.J., Reicheneder, C. et al. (2016) Efficacy of fluoride varnish for preventing white spot lesions and gingivitis during orthodontic treatment with fixed appliances—a prospective randomized controlled trial. *Clinical Oral Investigations,* 20, 2371–2378. <https://doi.org/10.1007/s00784-016-1730-6>.

Knösel, M., Forslund, L., Jung, K. and Ziebolz, D. (2012) Efficacy of different strategies in protecting enamel against demineralization during fixed orthodontic treatment. *Journal of Orofacial Orthopedics*, 73, 194-203.

Mehta, A., Paramshivam, G., Chugh, V.K., Singh, S., Halkai, S. and Kumar, S. (2015) Effect of light-curable fluoride varnish on enamel demineralization adjacent to orthodontic brackets: an in-vivo study. *American Journal of Orthodontics and Dentofacial Orthopaedics*, 148, 814-820.

Øgaard, B., Larsson, E., Henriksson, T., Birkhed, D. and Bishara, S.E. (2001) Effects of combined application of antimicrobial and fluoride varnishes in orthodontic patients. *American Journal of Orthodontics and Dentofacial Orthopaedics*, 120, 28-35. Erratum in: *American Journal of Orthodontics and Dentofacial Orthopaedics*, 2001, 120, 279. PMID: 11455374.

Perrini, F., Lombardo, L., Arreghini, A., Medori, S. and Siciliani, G. (2016) Caries prevention during orthodontic treatment: In-vivo assessment of high-fluoride varnish to prevent white spot lesions. *American Journal of Orthodontics and Dentofacial Orthopaedics*, 149, 238-243.

Poornima, P., Krithikadatta, J., Ponraj, R.R., Velmurugan, N. and Kishen, A. (2021) Biofilm formation following chitosan-based varnish or chlorhexidine-fluoride varnish application in patients undergoing fixed orthodontic treatment: a double blinded randomised controlled trial. *BMC Oral Health*, 23, 465.

Restrepo, M., Bussaneli, D.G., Jeremias, F., Cordeiro, R.C., Raveli, D.B., Magalhães, A.C., Candolo, C. and Santos-Pinto, L. (2016) Control of White Spot Lesions with Use of Fluoride Varnish or Chlorhexidine Gel During Orthodontic Treatment A Randomized Clinical Trial. *Journal of Clinical Pediatric Dentistry*, 40, 274-280.

Shah, M., Paramshivam, G., Mehta, A., Singh, S., Chugh, A., Prashar, A. and Chugh, V.K. (2018) Comparative assessment of conventional and light-curable fluoride varnish in the prevention of enamel demineralization during fixed appliance therapy: a split-mouth randomized controlled trial. *European Journal of Orthodontics*, 6, 132-139.

Shinaishin, S.F., Ghobashy, S.A. and El-Bialy, T.H. (2011) Efficacy of light-activated sealant on enamel demineralization in orthodontic patients: an atomic force microscope evaluation. *Open Dentistry Journal*, 5, 179-186.

Silva, V.M., Massaro, C., Buzalaf, M.A.R., Janson, G. and Garib, D. (2021) Prevention of non-cavitated lesions with fluoride and xylitol varnishes during orthodontic treatment: a randomized clinical trial. *Clinical Oral Investigations*, 25, 3421-3430.

Vivaldi-Rodrigues, G., Demito, C.F., Bowman, S.J. and Ramos, A.L. (2006) The effectiveness of a fluoride varnish in preventing the development of white spot lesions. *World Journal of Orthodontics*, 7, 138-144.
